# Individual socioeconomic status as a modifier of the association between high ambient temperature and hospital admissions: a time series study in Hong Kong, 2010–2019

**DOI:** 10.1007/s11356-022-20512-7

**Published:** 2022-05-06

**Authors:** Yi Tong Guo, William Bernard Goggins, Emily Ying Yang Chan, Kin Fai Ho

**Affiliations:** 1 The Jockey Club School of Public Health and Primary Care, The Chinese University of Hong Kong, Hong Kong Special Administrative Region, China; 2 Collaborating Centre for Oxford University and CUHK for Disaster and Medical Humanitarian Response (CCOUC), The Chinese University of Hong Kong, Hong Kong Special Administrative Region, China

**Keywords:** Climate change, Temperature, Heat, Hospital admissions, Morbidity, Effect modification, Socioeconomic status

## Abstract

Few studies have examined individual socioeconomic status (SES) as a potential modifier of ambient temperature–health associations, especially for temperature-related hospitalizations. We fit penalized distributed lag non-linear models within generalized additive models to study the short-term associations (0–3 days) between temperature and hospital admissions stratified by common causes, age, and individual SES, as determined by whether patients received public assistance (PA) to cover their medical fee at the time of hospitalizations, during the hot season (May 15 to October 15) in Hong Kong for the years 2010–2019. We calculated the ratio of relative risk (RRR) and corresponding 95% confidence interval (CI) to statistically test the difference of the associations between PA groups. For 75 + patients, the PA group had significantly increased risks of hospitalizations at higher temperature for most causes, with relative risks (RR, 99th %ile vs. 25%ile) and 95% CIs of 1.138 (1.099, 1.179), 1.057 (1.008, 1.109), and 1.163 (1.094, 1.236) estimated for all non-cancer non-external, circulatory, and respiratory admissions, respectively. There were slight decreases of RRs with higher temperature for 75 + patients without PA. The strengths of temperature–hospitalization associations were strongly and significantly different between PA groups for all examined causes for 75 + patients, with the most considerable discrepancy found for ischemic heart disease (RRR = 1.266; 95% CI, 1.137, 1.410). Hospitalizations for patients aged 15–74 were less affected by heat, and the difference of the associations between groups was small. Individual SES is a significant modifier of high temperature–hospitalization associations in Hong Kong among the elderly. Public health interventions are needed to better protect this subpopulation from adverse health impacts of high temperature.

## Background

High ambient temperature is a well-documented risk factor for health. Prior findings showed elevated risks of mortality and morbidity at high temperature in a wide range of locations (Basu 2009; Hajat et al. 2010; Ye et al. 2012; Gronlund et al. 2018; Green et al. 2019). Ongoing climate warming is expected to increase temperature-related health burden in the long run, and more scientific efforts are required to identify potentially vulnerable populations (Lee et al. 2019).

The strengths of temperature–health associations are heterogeneous across populations of varying characteristics (Hajat et al. 2010; Son et al. 2019). A recent review of effect modifiers on temperature-related mortality suggested moderate to strong evidence of higher heat risks with older age, pre-existing conditions, and low socioeconomic status (SES) (Son et al. 2019). People of low SES might suffer from inequality of response options or insufficiency of adaptive capacity to climate change (IPCC 2014). Some multisite studies examined the modifying effects via including site-specific socioeconomic characteristics as meta-predictors for temperature–mortality associations (Zanobetti et al. 2013; Chung et al. 2017; Sera et al. 2019). Alternatively, a few studies stratified the temperature–mortality associations by single indicators of individual SES (e.g., education level, occupational class) (Medina-Ramón et al. 2006; Huang et al. 2015) or used neighborhood socioeconomic indicators or comprehensive indices as a proxy to one’s actual SES in subgroup analyses (Chan et al. 2010; Wichmann et al. 2012; Liu et al. 2020). However, socioeconomic factors could be related to each other and to demographic factors (e.g., age, gender) (Son et al. 2019), and none of these studies considered possible interplay between the modifiers. In addition, most prior studies investigated modifying effects of SES on temperature-related mortality; less was known about how temperature disproportionately affected hospitalizations among individuals with different SES. A South Korea study considered types and premium of medical insurance as indicators of individual SES, where people with Medicaid, the insurance provided for the low-income brackets, and low premium were seen as the low SES groups (Kwon et al. 2015). This study has found the low SES groups were at higher heat and cold risks for acute myocardial infarction (AMI) hospitalizations. However, it solely focused on one specific cause and was conducted in temperate zone (Kwon et al. 2015).

Hong Kong, a highly developed metropolis of China, has had the highest wealth disparity among all developed countries and regions (Oxfam Hong Kong 2018; UNDP 2020). The city has a subtropical climate, where the hot and humid summer could be long persisting from May to October (Hong Kong Observatory 2021). It is of particular concern about the extent to which people living with socioeconomic disadvantages have been negatively affected by extreme heat in the city. Our past research in Hong Kong reported an inverse gradient relationship between area-level SES and heat-related mortality (Chan et al. 2010; Liu et al. 2020).

Accordingly, we aimed to assess modifying effects of individual SES, i.e., whether or not patients were receiving public assistance (PA) to cover their medical expenses, on the short-term associations between high ambient temperature and cause–age-specific hospital admissions during the hot season in Hong Kong. We modeled the potentially non-linear and delayed effects of temperature by fitting penalized distributed lag non-linear model (DLNMs), which were a recently improved version of DLNMs allowing built-in smoothness selection of marginal functions and flexible accommodation of assumptions on the lag–response association (Gasparrinia et al. 2010; Gasparrini et al. 2017).

## Methodology

### Hospital admission data

Hospital admissions via accident and emergency department during 2010–2019 were aggregated on a daily basis and obtained from the Hong Kong Hospital Authority, which administers a citywide database covering admission records from all public medical sectors (Hong Kong Hospital Authority 2021a). The study was limited to the hot season, May 15 to October 15, of each year, to control for potential seasonal bias. Patients with unknown age or not yet discharged from hospital at the date of data extraction were not included. Planned admissions were not included as they were unlikely to be affected by meteorological conditions. Counts of 1–4 were suppressed in the original data set and thus imputed as 4 for subsequent analyses.

Daily counts of admissions were cross-classified by cause, age (15–74, 75 +) and PA status (no, yes). Cause groups of interest and the corresponding codes in the International Statistical Classification of Diseases and Related Health Problems, Ninth Revision, Clinical Modification (ICD-9-CM) were as follows: all non-cancer non-external (001–139, 240–799), circulatory (390–459), ischemic heart disease (IHD, 410–414), respiratory (460–519), chronic obstructive pulmonary disease (COPD) and asthma (490–496), and pneumonia and influenza (480–487).

PA is government-oriented and granted to people with financial difficulties to waive their payment of public medical fees (Hong Kong Hospital Authority 2021b). Eligible recipients are those having little income or asset, including recipients of three social welfare schemes (i.e., recipients of Comprehensive Social Security Assistance, Level 0 Voucher holders of the Pilot Scheme on Residential Care Service Voucher for the Elderly, and Higher Old Age Living Allowance recipients aged 75 or above) and three vulnerable groups (i.e., the low-income group, chronically ill patients, and elderly patients living in poverty) (Hong Kong Hospital Authority 2021b). PA status is, therefore, a noteworthy indicator of individual SES. In this study, we determined patients receiving PA as the low SES group and those not receiving PA as the high SES group.

### Weather and air pollutant data

Daily data of meteorological variables, including mean temperature (${}^{\circ }$ C), mean relative humidity (RH, %), mean wind speed (km/h), and total rainfall (mm), were obtained for the same period from the Hong Kong Observatory weather station, which is located in the center of the city (Tsim Sha Tsui area). Daily mean concentrations of air pollutants ($\mu$ g/m^3^), including fine suspended particulates (PM_2.5_), ozone (O_3_), nitrogen dioxide (NO_2_), and sulfur dioxide (SO_2_), were collected from 14 of the 15 general stations belonging to the Environmental Protection Department and were then averaged across stations. Data from Tap Mun station, located in a remote area, was excluded as it may not represent the general population.

### Statistical analyses

We first fit penalized DLNMs (Gasparrinia et al. 2010; Gasparrini et al. 2017) for daily mean temperature by specifying a cross-basis function of P-splines (PS) with 5 *degree of freedom* (*df*) for each dose–response and lag–response dimension. We determined a lag period of 0–3 days as heat effects were found to be immediate and occurred within a few days (Basu 2009). While the dose–response relationship was penalized by the default second-order difference penalty of the PS smoother, we performed doubly varying penalties on the lag–response relationship, i.e., a varying difference penalty to allow different degrees of smoothness along the lag structure, plus a varying ridge penalty to shrink different parts of the lag structure towards the null value (Gasparrini et al. 2017). It was assumed that temperature effects on health were smoother and approaching the null value at longer lags (Gasparrini et al. 2017).

Next, we regressed daily counts of hospital admissions by including the cross-basis function of temperature as a parametric term in quasi-Poisson generalized additive models (GAMs) (Wood 2006) and controlled for the following covariates with specific choices: thin-plate regression splines of day of the study with a maximum of 10 *df* to account for time trend; thin-plate regression splines of day of the year with a maximum of 3 *df* to account for seasonality; indicator variables for day of the week, public holidays, and typhoon days (signal 8 and above) to account for weekly, holiday, and heavy weather patterns, respectively; a cross-basis function of RH with similar specification to the temperature’s; and thin-plate regression splines of square-root transformed environmental variables (i.e., wind speed, total rainfall, PM_2.5_, O_3_, and NO_2_) with a maximum of 2 *df*, respectively. Same-day rainfall was included given the presuppositions that it might prevent patients with mild symptoms from visiting the hospital (Chan et al. 2013). Environmental covariates were modeled using smooth functions to allow automatic variable selection and minimal curvature as their effects were approximately linear (Wood et al. 2016; Abed Al Ahad et al. 2020). Square-root transformation was to reduce impacts of outliers. Thin-plate regression splines were adopted for being the optimal smoothers of any given basis dimension (Wood 2003). The choice of 1 *df* per year for trend was used in our previous studies (Chan et al. 2018; Lam et al. 2018b). We determined restricted maximum likelihood (REML) method for smoothness selection for its good inferential properties (Wood 2011). Automatic variable selection was applied by specifying an argument “select = TRUE” in the GAMs, which added an extra penalty to each smooth and penalized only functions in the null space of its original penalty to zero (Wood 2006; Wood et al. 2016).

We estimated the temperature–hospitalization associations by computing the cumulative relative risk (RR), summed up across the lag period and centered at the 25th percentile of the temperature distribution, and corresponding 95% confidence interval (95% CI). RRs for specific temperature percentiles were also reported, with moderate heat effects defined as RRs for the 90th percentile (30.2C) and extreme heat effects defined as RRs for the 99th percentile (31.1C). The difference between PA groups was statistically tested by computing the ratio of relative risk (RRR) and its 95% CI comparing the PA group to the no-PA group (Altman 2003). Subgroup analyses were performed for each cause–age–PA group. We ran sensitivity analyses to test the robustness of the main results by varying the types of basis functions and choices of *df* or dimensions. All analyses were done in the statistical environment R version 4.1.1 (R Core Team 2021) using dlnm (Gasparrini and Armstrong 2011) and mgcv (Wood 2006) packages.

## Result

Descriptive statistics of environmental variables and hospital admissions were shown in Table 1 and Table 2, respectively. The daily mean temperature ranged from 20.5 to 32.4 °C and had a median (interquartile range, IQR) of 28.7 °C (27.3–29.7 °C) during the study period. The weather was humid in the summer, with a median daily mean RH of 80% (IQR 76–85%). Around 45% of the 75 + patients received PA, while this proportion was lower (17.4%) for the 15–74 group.

**Table 1:** Descriptive statistics of meteorological variables and air pollutants in the hot season (May 15 to October 15), Hong Kong, 2010–2019

Variable	Mean (SD)	Min	25th	Median	75th	Max
Mean temperature (°C)	28.4 (1.7)	20.5	27.3	28.7	29.7	32.4
Mean RH (%)	80.4 (7.7)	40.0	76.0	80.0	85.0	98.0
Mean wind speed (km/h)	21.1 (10.3)	4.4	13.4	19.4	27.0	102.1
Total rainfall (mm)	11.4 (26.4)	0.0	0.0	0.4	10.2	273.6
Mean PM_2.5_ (μg/m^3^)	18.4 (12.9)	4.3	9.9	13.4	22.4	78.1
Mean O_3_ (μg/m^3^)	39.1 (26.4)	6.5	19.9	29.5	51.9	164.9
Mean NO_2_ (μg/m^3^)	43.0 (15.9)	4.1	31.7	39.8	50.1	124.2
Mean SO_2_ (μg/m^3^)	9.7 (5.2)	3.3	6.0	8.3	12.1	45.3

Abbreviation: RH, relative humidity; PM2.5, fine particulate matter; O3, ozone; NO2, nitrogen dioxide; SO2, sulfur dioxide; SD, standard deviation; Min, the minimum of the distribution; 25th, the 25th percentile of the distribution; 75th, the 75th percentile of the distribution; Max, the maximum of the distribution.

**Table 2:** Descriptive statistics of hospital admissions in the hot season (May 15 to October 15), Hong Kong, 2010–2019

Subgroup	No PA			PA	
	Total N (%)	Mean daily count (SD)		Total N (%)	Mean daily count (SD)
Non-cancer non-external					
15–74	1,045,304 (82.6)	678.8 (98.0)		220,035 (17.4)	142.9 (16.1)
75 +	512,965 (55.1)	333.1 (87.9)		417,789 (44.9)	271.3 (114.1)
Circulatory					
15–74	123,714 (84.8)	80.3 (14.6)		22,158 (15.2)	14.4 (4.2)
75 +	91,324 (60.6)	59.3 (17.7)		59,306 (39.4)	38.5 (19.2)
IHD					
15–74	22,375 (79.7)	14.5 (4.0)		5,714 (20.3)	3.7 (1.5)
75 +	15,508 (61.6)	10.1 (4.4)		9,677 (38.4)	6.3 (2.8)
Respiratory					
15–74	95,439 (73.5)	62.0 (15.6)		34,327 (26.5)	22.3 (6.0)
75 +	100,592 (49.7)	65.3 (23.6)		101,667 (50.3)	66.0 (21.8)
COPD and asthma					
15–74	27,814 (66.6)	18.1 (4.9)		13,930 (33.4)	9.0 (3.2)
75 +	30,676 (51.6)	19.9 (8.3)		28,734 (48.4)	18.7 (6.9)
Pneumonia and influenza					
15–74	36,337 (72.6)	23.6 (10.0)		13,680 (27.4)	8.9 (3.7)
75 +	52,386 (47.5)	34.0 (14.1)		57,828 (52.5)	37.6 (13.7)

Abbreviation: PA, public assistance; SD, standard deviation; COPD, chronic obstructive pulmonary disease; IHD, ischemic heart disease.

**Fig. 1:**
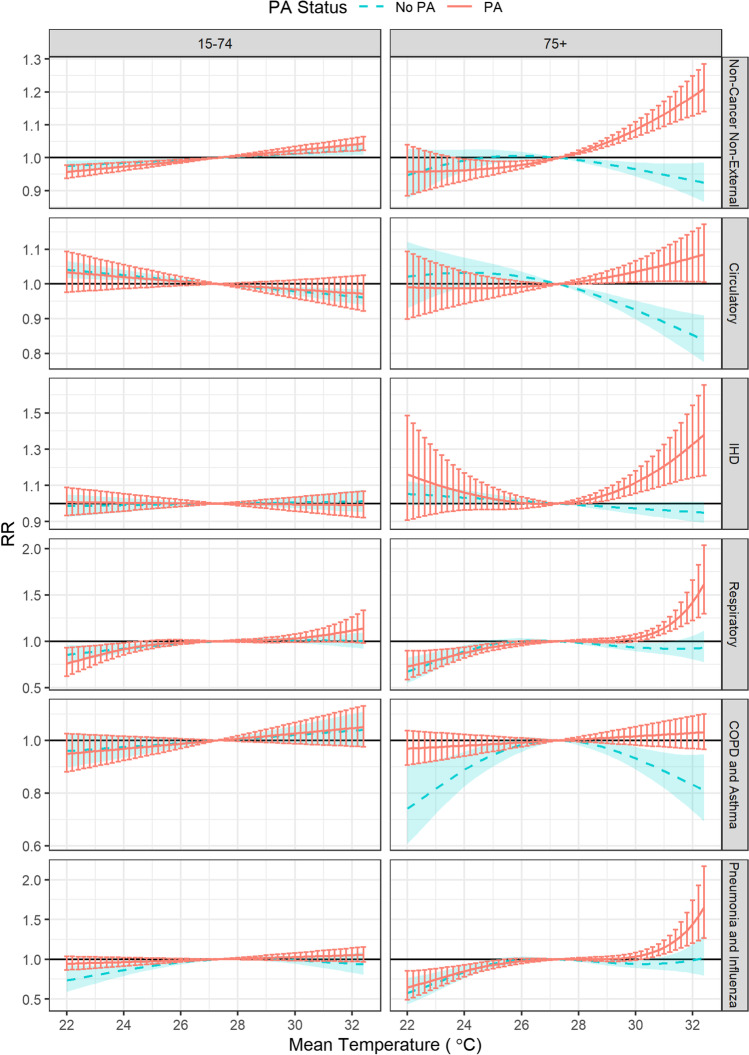
The cumulative associations (0–3 days) of hospital admissions with temperature in subgroups in the hot season (May 15 to October 15), Hong Kong, 2010–2019. RRs were in dashed/solid lines, and 95% CIs were in shaded areas/error bars. Abbreviation: PA, public assistance; COPD, chronic obstructive pulmonary disease; IHD, ischemic heart disease; RR, relative risk centered at 27.3 ${}^{\circ }$ C; CI, confidence interval

Results of the cumulative associations (0–3 days) between high temperature and hospitalizations stratified by cause, age, and PA status were plotted in Fig. 1, and comparisons of RRs between PA groups were summarized in Table 3. RRs of heat-related hospitalizations were strongly and statistically different between PA groups for all examined causes for 75 + patients. Specifically, the PA group had significantly elevated risks associated with extreme heat (99th vs. 25th) for total non-cancer non-external (RR = 1.138; 95% CI, 1.099–1.179), circulatory (RR = 1.057; 95% CI, 1.008–1.109), and respiratory (RR = 1.163; 95% CI, 1.094–1.236) admissions, whereas the no-PA group had significantly decreased risks of hospitalizations when exposed to higher temperature. The most evident discrepancy between PA groups was observed for IHD admissions (RRR = 1.266; 95% CI, 1.137–1.410) among 75 + patients. Moreover, the largest estimate of heat RRs was found for IHD admissions for 75 + patients with PA (RR = 1.219; 95% CI, 1.106–1.344). Although associations of COPD and asthma admissions among 75 + patients were significantly modified by PA status (RRR = 1.164; 95% CI, 1.056–1.279), heat risks were estimated to marginally increase for the PA group (RR = 1.023; 95% CI, 0.975, 1.074) and significantly decrease for the no-PA group (RR = 0.879; 95% CI, 0.811–0.953). Hospitalizations among 15–74 patients were generally less affected by high temperature, and the difference of heat RRs between PA groups was small. High temperature slightly increased non-cancer non-external admissions among 15–74 patients for both PA groups, with stronger effects for the PA group (RR = 1.032; 95% CI, 1.017–1.048) than the no-PA group (RR = 1.018; 95% CI, 1.007–1.029).

**Table 3:** Comparison of RRs at moderate (90th %ile) and extreme (99th %ile) heat between PA groups in the hot season (May 15 to October 15), Hong Kong, 2010–2019

Subgroup	Heat^1^	No PA			PA		RRR	95% CI for RRR
		RR	95% CI		RR	95% CI		
Non-cancer non-external								
15–74	Moderate	1.013^*^	(1.005, 1.022)		1.024^*^	(1.013, 1.036)	1.011	(0.996, 1.025)
	Extreme	1.018^*^	(1.007, 1.029)		1.032^*^	(1.017, 1.048)	1.014	(0.995, 1.033)
75 +	Moderate	0.962^*^	(0.939, 0.986)		1.095^*^	(1.069, 1.121)	1.138^*^	(1.100, 1.177)
	Extreme	0.947^*^	(0.913, 0.982)		1.138^*^	(1.099, 1.179)	1.202^*^	(1.142, 1.265)
Circulatory								
15–74	Moderate	0.978^*^	(0.965, 0.991)		0.984	(0.956, 1.012)	1.006	(0.974, 1.038)
	Extreme	0.971^*^	(0.955, 0.988)		0.979	(0.942, 1.017)	1.008	(0.966, 1.051)
75 +	Moderate	0.918^*^	(0.889, 0.948)		1.039^*^	(1.006, 1.073)	1.132^*^	(1.082, 1.184)
	Extreme	0.887^*^	(0.845, 0.930)		1.057^*^	(1.008, 1.109)	1.192^*^	(1.114, 1.276)
IHD								
15–74	Moderate	1.007	(0.973, 1.043)		0.996	(0.955, 1.038)	0.988	(0.936, 1.044)
	Extreme	1.010	(0.965, 1.057)		0.994	(0.941, 1.050)	0.985	(0.917, 1.058)
75 +	Moderate	0.972	(0.938, 1.006)		1.133^*^	(1.064, 1.205)	1.166^*^	(1.086, 1.252)
	Extreme	0.963	(0.920, 1.008)		1.219^*^	(1.106, 1.344)	1.266^*^	(1.137, 1.410)
Respiratory								
15–74	Moderate	1.013	(0.979, 1.048)		1.037	(0.990, 1.086)	1.024	(0.966, 1.085)
	Extreme	1.009	(0.959, 1.063)		1.069	(0.993, 1.152)	1.059	(0.968, 1.159)
75 +	Moderate	0.931^*^	(0.887, 0.978)		1.049^*^	(1.009, 1.091)	1.126^*^	(1.058, 1.199)
	Extreme	0.921^*^	(0.855, 0.993)		1.163^*^	(1.094, 1.236)	1.262^*^	(1.146, 1.390)
COPD and asthma								
15–74	Moderate	1.022	(0.985, 1.061)		1.029	(0.986, 1.073)	1.006	(0.951, 1.064)
	Extreme	1.029	(0.981, 1.080)		1.038	(0.982, 1.096)	1.008	(0.937, 1.084)
75 +	Moderate	0.923^*^	(0.877, 0.971)		1.017	(0.981, 1.056)	1.102^*^	(1.035, 1.174)
	Extreme	0.879^*^	(0.811, 0.953)		1.023	(0.975, 1.074)	1.164^*^	(1.059, 1.279)
Pneumonia and influenza								
15–74	Moderate	0.986	(0.933, 1.041)		1.032	(0.981, 1.084)	1.047	(0.972, 1.127)
	Extreme	0.965	(0.887, 1.050)		1.042	(0.976, 1.112)	1.080	(0.970, 1.201)
75 +	Moderate	0.941	(0.884, 1.001)		1.048	(0.997, 1.102)	1.114^*^	(1.028, 1.206)
	Extreme	0.950	(0.863, 1.045)		1.168^*^	(1.079, 1.265)	1.230^*^	(1.086, 1.393)

^1^Moderate heat, the 90th percentile of temperature, 30.2 °C; extreme heat, the 99th percentile of temperature, 31.1 °C; ^*^p < 0.05

Abbreviation: PA, public assistance; RR, cumulative relative risk (0-3 days) with reference to 27.3ºC; CI, confidence interval; RRR, ratio of relative risk comparing with PA to without PA; COPD, chronic obstructive pulmonary disease; IHD, ischemic heart disease

## Discussion

In this 10-year time series study, we investigated the modifying effects of individual SES, based on whether a patient was receiving PA at the time of hospitalizations, on the short-term associations between high ambient temperature and cause–age-specific hospital admissions in a subtropical urban setting. For the elderly aged 75 + , the lower SES (PA) group had significantly elevated risks of hospitalizations for all examined causes (except COPD and asthma) with higher temperature. In comparison, slight decreases in hospitalization risks at higher temperature were observed for the higher SES (no PA) group. The modification of heat–hospitalization associations by individual SES was substantial and statistically significant for the elderly, with the most considerable discrepancy found for IHD admissions. In contrast, the between-SES group difference was small for patients aged 15–74. While this younger population was less affected by summer heat, we found slight, but statistically significant, increases in heat risks for non-cancer non-external admissions for both SES groups.

Several factors could be underlying the different susceptibility to heat between SES groups, especially for the elderly, in the setting of Hong Kong. People who receive PA have socioeconomic disadvantages evaluated by the government as having little income and assets (Hong Kong Hospital Authority 2021b). They may have no air conditioner/conditioning (AC) in their residence or seldom use AC in the summer because of the high cost of electricity. Disadvantaged socio-demographic groups also tend to live in the neighborhood with intense urban heat island effects, which could exacerbate adverse high temperature impacts on health (Goggins et al. 2012; Wong et al. 2016). The long and hot summer in Hong Kong may have exceeded the capacity of people in lower SES to cope and thus becomes a risk factor for a range of adverse health outcomes in this population.

A review of temperature-related mortality has suggested higher heat risks associated with low community-level SES (Son et al. 2019). Our mortality studies in Hong Kong have found that people living in low socioeconomic districts were more vulnerable to heat effects (Chan et al. 2010; Liu et al. 2020). Effect modifications of SES on temperature–hospitalization associations were less studied. A study in New York City has found that patients living in the neighborhoods with lower SES were at higher heat risks for cardiovascular and respiratory hospitalizations (Lin et al. 2009). In contrast, a study in Copenhagen, Denmark, has reported no significant modification of heat-related AMI hospitalizations by neighborhood SES (Wichmann et al. 2012). Our findings for IHD hospitalizations showed heat effects were pronounced for the elderly with lower SES and marginal for other subgroups, which were comparable to the results from a South Korea study of temperature–AMI hospitalization associations (Kwon et al. 2015). The South Korea study examined individual SES modifiers and found the low SES groups (Medicaid and low health insurance premium), as well as the elderly, had higher same-day risks for heat-related AMI admissions (Kwon et al. 2015). Our study was the first to compare associations of high temperature with respiratory hospitalizations between adults with different individual SES. We found an overall positive and strong association for hospitalizations due to respiratory diseases and pneumonia and influenza for the elderly with lower SES and insignificant heat effects for those with higher SES. Results for COPD and asthma hospitalizations among the elderly showed a different pattern, as high temperature had a significant protective effect for the higher SES group, while it slightly but insignificantly increased admissions for the lower SES group. Nevertheless, our previous studies in Hong Kong found COPD admissions (Lam et al. 2018a) were generally not sensitive to heat, and risks of asthma admissions (Lam et al. 2016) significantly increased with higher temperature. A decreasing trend of heat-related risks of hospitalizations was also observed for other causes for the elderly with higher SES. Only about 2% of the 75 + population keep working in Hong Kong (Chan and Yip 2019), and it is possible that the elderly with higher SES avoid going outdoors during the heat and could afford AC.

Our findings that the elderly with lower SES were more vulnerable to heat-related hospitalizations provide some public health implications for policymakers and the scientific community. As summer temperature keeps rising in climate change, there is an urge to spread awareness of adverse heat impacts on health. Adaption plans should be carried out locally to mitigate the impacts and reduce population vulnerability, such as vouchers for AC or electricity use and social workers’ check on the elderly during high temperature.

Our study has several strengths. First, we have individual SES data, which was unavailable in many prior studies. We statistically quantify the difference of high temperature effects on hospital admissions due to a range of causes between SES groups and consider the interaction between individual SES and age by running the analysis within each age group, which was less examined previously. Second, we have relatively long time series data spanning 10 years. Finally, we apply technically more advanced statistical methodology, which provides improved inferential properties. A limitation, by the nature of routine data, is that levels of meteorological variables and air pollutants were measured outdoor at fixed monitoring stations which may not represent actual individual exposure.

## Conclusion

Individual SES is a significant modifier of the associations between high temperature and hospital admissions among the elderly in Hong Kong. The elderly in lower SES has elevated risks for non-cancer non-external, respiratory, pneumonia and influenza, circulatory, and IHD hospitalizations with higher temperature. Public health interventions are needed to better protect this group from adverse health impacts of high temperature.

## Data Availability

The data of meteorological variables are available in the Hong Kong Observatory open database, https://www.hko.gov.hk/en/abouthko/opendata_intro.htm. The data of air pollutants are available in the Hong Kong Environmental Protection Department open database, https://cd.epic.epd.gov.hk/EPICDI/air/station/?lang=en. The data of hospital admissions are available from the Hong Kong Hospital Authority, but restrictions apply to the availability of these data, which were used under license for the current study, and so are not publicly available.

## Abbreviations

SES: Socioeconomic status
PA: Public assistance
DLNM: Distributed lag non-linear model
ICD-9-CM: International Statistical Classification of Diseases and Related Health Problems, Ninth Revision, Clinical Modification
COPD: Chronic obstructive pulmonary disease
IHD: Ischemic heart disease
RH: Relative humidity
PM_2.5_: Fine suspended particulates
O_3_: Ozone
NO_2_: Nitrogen dioxide
SO_2_: Sulfur dioxide
df: Degree of freedom
GAM: Generalized additive model
REML: Restricted maximum likelihood
RR: Relative risk
CI: Confidence interval
RRR: Ratio of relative risk
AC: Air conditioning
AMI: Acute myocardial infarction
